# Role of Neutrophil CD64 Index as a Screening Marker for Late-Onset Sepsis in Very Low Birth Weight Infants

**DOI:** 10.1371/journal.pone.0124634

**Published:** 2015-04-20

**Authors:** Florian Kipfmueller, Jessica Schneider, Julia Prusseit, Ioanna Dimitriou, Berndt Zur, Axel R. Franz, Peter Bartmann, Andreas Mueller

**Affiliations:** 1 Department of Neonatology and Pediatric Critical Care, University Children‘s Hospital Bonn, Bonn, Germany; 2 Department of Medical Biometry, Informatics and Epidemiology, University Hospital Bonn, Bonn, Germany; 3 Institute for Clinical Chemistry and Clinical Pharmacology, University Hospital Bonn, Bonn, Germany; 4 Current affiliation: Department of Neonatology, University Children’s Hospital Tuebingen, Tuebingen, Germany; University of Florida, UNITED STATES

## Abstract

**Introduction:**

The role of CD64 in late onset sepsis (LOS) in preterm infants has been described in several studies. Aim of this study was to investigate whether CD64 expression is increased in the days before clinical manifestation of LOS.

**Methods:**

Patients with birth weight below 1,500g were eligible for study participation. During routine blood sampling CD64 index was determined between day of life 4 and 28. Patients were allocated to one of four groups: (1) blood-culture positive sepsis, (2) clinical sepsis, (3) symptoms of infection without biochemical evidence of infection, or (4) patients without suspected infection. Kinetics of CD64 expression were compared during a period before and after the day of infection in the respective groups.

**Results:**

50 infants were prospectively enrolled and allocated to each group as follows: group (1) n = 7; group (2) n = 10; group (3) n = 8; and group (4) n = 25. CD64 index was elevated in 57% of patients in group (1) at least two days before infection. In contrast only 20% in the clinical sepsis group and 0% in group (3) had an elevated CD64 index in the days before infection. 10 of the 25 patients in the control group (4) presented increased CD64 index values during the study period.

**Conclusions:**

The CD64 index might be a promising marker to detect LOS before infants demonstrate signs or symptoms of infection. However, larger prospective studies are needed to define optimal cut-off values and to investigate the role of non-infectious inflammation in this patient group.

## Introduction

Very low birth weight (VLBW, i.e. birth weight below 1,500g) infants are at increased risk to suffer from infection or late onset sepsis (LOS, i.e. sepsis after 72 hours of life) during their hospital stay mainly because of their immature immune system, a high rate of invasive procedures (e.g. venous or arterial lines, punctures, drainages) and their dependence on either invasive or non-invasive respiratory support [1]. Despite improvement in early diagnosis and management, late onset sepsis in VLBW infants remains a serious condition with high morbidity and mortality. Incidence rates for VLBW infants as high as 24.5% in a large NICHD network analysis have been reported [2]. A recent report by Hornik and coworkers revealed an incidence of LOS in this population of 12.2% [3]. Of notice, 50% of the 99,796 VLBW infants in Hornik’s study had at least one culture obtained for suspected LOS. A recent report of the German national surveillance system for nosocomial infection in infants (NEO-KISS) with a birth weight below 1,000g, and between 1,000g and 1,499g demonstrated an incidence for blood-culture positive sepsis of 23% and 9%, respectively [4]. However, considerably more infants are treated with antibiotics for suspected infection during their hospital stay due to unspecific symptoms and a lack of accurate biomarkers for sepsis.

Küster et al. measured daily blood cytokine levels during routine lab testing in 101 VLBW infants in the first 28 days of life [5]. It was demonstrated that interleukin-6 (IL-6) and interleukin-1 receptor antagonist were already increased 1–2 days before the clinical diagnosis of sepsis. The high amount of blood necessary for daily cytokine level determination withheld this approach to become part of standard clinical practice such as point-of-care blood gas analysis. With a similar study design as used by Küster and coworkers, Lam et al investigated the potential of neutrophil CD64 as a surveillance biomarker [6]. Daily measurements of CD64 expression detected LOS/Necrotizing enterocolitis (NEC) 1.5 days before infants presented signs of infection.

After recognition of a foreign antigen, opsonins such as immunoglobulin G (IgG) or C3b complement component bind the antigen and form an IgG-antibody complex. The Fc fragment of the IgG molecule contacts with specific neutrophil cell surface receptors, such as the Fc-gamma receptors. The interaction of the Fc fragment and the Fc-gamma receptors initiate processes of phagocytosis, degranulation and antibody-dependent cellular cytotoxicity. CD64, also known as Fc-gamma receptor 1 (FcγR1), is a high affinity receptor, extensively expressed on the surface of neutrophil granulocytes during bacterial infection [7,8,9]. Cross-linking of IgG and CD64 promotes the submembranous activation of contractile microfilaments, resulting in the formation of moving pseudopods. Pseudopods are crucial for the ingestion of foreign particles before degranulation is initiated [10]. Fjaertoft and coworkers reported that CD64 expression in preterm infants is similar to that in term neonates, infants, children, and adults [11]. CD64 expression can be measured by flow cytometry with small amounts of blood [12]. The role of CD64 in early-onset sepsis (EOS) and LOS in term and preterm infants has been investigated in the past with promising results, especially for the combination with other infection markers [13,14,15,16,17,18,19,20]. The calculation of a CD64 index has been postulated by some investigators to reduce bias arising from manual methods [21,22]. However, no study has demonstrated the role of CD64-index determination as a potential marker for early diagnosis of LOS in the absence of suspicious clinical signs.

The purpose of this feasibility study was to investigate the kinetics of CD64 expression before and during culture proven sepsis compared to clinical sepsis and suspected infection in VLBW infants between postnatal day 4 and day 28 and to test the set-up for a larger prospective trial.

## Materials and Methods

### Ethical statement

The study was approved by the Bonn University Hospital Institutional Review Board. Written informed consent was obtained from parents or legal representatives before enrollment in the study.

### Study population

All VLBW infants admitted to the neonatal intensive care unit (NICU) of the University of Bonn between September 2009 and June 2010 were eligible for participation, apart from infants with congenital malformation and infants receiving palliative care. Patients were not excluded for the presence of EOS. Blood specimens for CD64 analysis were collected during routine sampling for hematological or biochemical evaluation or blood gas analysis between postnatal day 4 and 28. In case of a suspected infection a complete blood count, C-reactive Protein (CRP) and IL-6 were determined. At least one aerobic blood culture was inoculated before start of antibiotic treatment. Antibiotic treatment was not restricted by study protocol and was started whenever the medical team suspected infection.

### Classification of patients

Group allocation for infants with sepsis or NEC (using the modified Bell’s criteria [23]) was performed based on the result of the blood cultures (i.e. infants with NEC and a positive blood culture were allocated to the culture positive group; infants with NEC and a negative blood culture were allocated to group 2). Four patient groups were defined prospectively:

1. Culture positive group: Infants with clinical signs of sepsis or NEC and with at least one positive blood culture.
2. Clinical sepsis group: patients with signs and symptoms of sepsis or NEC, antibiotic treatment for at least 5 days and a negative blood culture. All patients had to fulfill at least two of the following criteria for diagnosis of sepsis for VLBW infants, published by the national institute for surveillance of nosocomial infection in Germany (Neo-KiSS) [24]: fever (>38.0°C), hypothermia (<36.5°C), temperature instability, tachycardia (>200 bpm), bradycardia (<80 bpm), abnormal capillary refilling time, pale skin color, apnea, metabolic acidosis (base deficit >10mmol/l), hyperglycemia (>140 mg/dl), increasing oxygen demand or elevated values of CRP (>10mg/l) or Interleukin-6 (>100pg/ml).
3. Indeterminate group: Infants with the suspicion of sepsis in whom treatment with antibiotics was started, but was discontinued early (i.e. <5d) due to judgment by the treating physician during therapy that infection retrospectively seemed unlikely.
4. Control group: Patients without episodes of sepsis or suspected sepsis during the study period.

Patients were allocated to one of the four groups by an investigator blinded to CD64 index results. CD64 index measurements were not accessible for the treating physician.

### CD64 index

Flow cytometry was accomplished on a FACSCalibur flow cytometer (Becton Dickinson, NY, USA) with 50μl EDTA-anticoagulated whole blood. Cell type differentiation was determined using the right-angle side scatter and forward scatter method. CD64 and CD163 expression on neutrophils, monocytes and lymphocytes were measured using the Leuko64 test kit (Trillium Diagnostics, ME, USA). The Leuko64 kit consists of three murine monoclonal antibodies with specificities to either CD64 (fluorescin isothiocynate (FITC) conjugated clones 22 and 32.2) or CD163 (phycoerythrin (PE) conjugated clone Mac2-148) and fluorescent standard beads.

According to manufacturer’s instruction whole blood was mixed with antibodies and incubated for 10 minutes at room temperature in the dark. Red cell lysis was performed afterwards using the kits ammonium-chloride-based lysis solution. The standard-beads suspension was added and flow-cytometry was performed on at least 50,000 leukocytes. Cells were identified based on their logarithmic side scatter dot-plot profiles. A gate was set around the different cell populations and the fluorescent intensity within the gate was analyzed using the CellQuest Software (Becton Dickinson, NY, USA). Mean fluorescent intensity (MFI) was defined as the geometric mean of the logarithmic fluorescence intensity emitted by the respective cell lines. The expression level of CD64 was measured as geometric MFI on neutrophils (positive for CD64 expression), monocytes (positive control, CD64 and CD163 expression), lymphocytes (negative control, no CD64 expression) and standard-beads (FITC and PE expression). The CD64 index was calculated using the ratio of the CD64-MFI of neutrophil granulocytes to that of the standard-beads using the Leuko64 QuantiCALC-Software (Trillium Diagnostics (Brewer, ME, USA). A cut-off value of 1.86 for CD64 index was used because this has been demonstrated in a previous study to be a good indicator for infection [25]. Due to the relatively small sample size for each day we did not calculate individual cut-off values for our study.

### Inflammation in asymptomatic infants (control group)

As an incidental finding an elevated CD64 index (> 1.86) was detected in several infants in the control group. These episodes were described as asymptomatic/non-infectious inflammation. An episode of inflammation was defined as the time between the first and the last day of elevated CD64 index. Increased CD64 index values following a previous episode of inflammation were described as a new episode. To reflect immune activation resulting from maternal infection at the time of delivery, episodes of inflammation were divided in early (i.e. first increased CD64 index on day of life 4 and 5) and late (i.e. beginning at day of life 6 or later) episodes. Episodes were correlated with maternal inflammation, preterm premature rupture of membranes (pPROM), peak CRP values during the first 10 days of life in respective infants, presence of mechanical ventilation and supplemental oxygen. Maternal inflammation was defined as either increased white blood count (> 10.3 G/l) or increased CRP (> 10 mg/l) and clinical signs of infection in mothers within 3 days before and after delivery, as documented by the obstetrics team. Patient charts and study documentation sheets were reviewed regarding presumable symptoms of infection.

### Study period

The individual study period was between postnatal day 4 and 28. To detect changes in inflammatory markers we defined an observational period of 10 days whenever sepsis or suspicion of sepsis occurred, similar to the study design used by Küster and colleagues [5]. Briefly, day 0 was the day of clinical diagnosis. The 4 days before day 0 were termed as day -4 to day -1, and the days following day 0 as day +1 to day +5. We compared each value during the 10 study days with an individual baseline value to assess an increase in CD64 index. The baseline value was the most recent value before the first day of the observation period (before day -4), when there was neither clinical suspicion nor objective evidence of sepsis. To avoid over- or underestimation of individual baseline values, a group-specific baseline value was not calculated for infants without any value before day -4.

### Statistics

Data were analyzed by SPSS for Windows (Release 15, SPSS Inc., Chicago, IL, USA). For comparison of demographic data of the four groups (culture positive, clinical sepsis, indeterminate and control group) the Kruskal-Wallis test and χ^2^ test were used.

Comparison of median CD64 index values on each day during the observation period within the respective groups was performed with the Mann-Whitney test. The Wilcoxon signed rank test was used to detect significant differences on each day compared to the baseline value. Sensitivity, specificity and 95% confidence interval was calculated for the cut-off value of 1.86.

## Results

**Fig 1:**
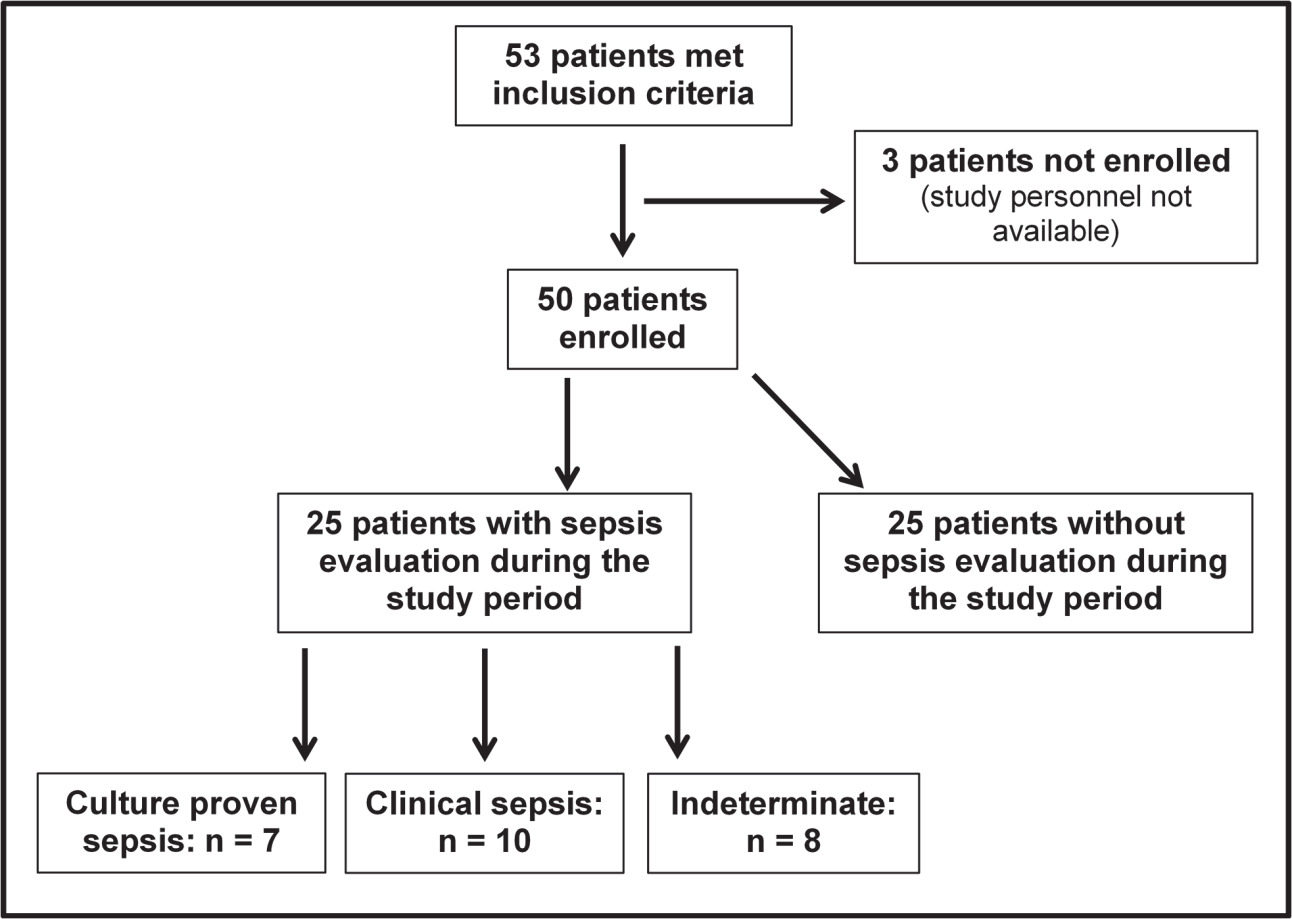
Flow diagram of group allocation of the 50 infants enrolled in the study. During the study period (i.e. day of life 4 until day of life 28), 25 infants were evaluated for sepsis. Infants with septicemia represent the culture positive group (n = 7). Infants with clinical features of infection, a negative blood culture and antibiotic treatment for at least 5 days were allocated to the clinical sepsis group (n = 10) and infants with evaluation for sepsis who were subsequently proven not to be infected were allocated to the indeterminate group (n = 8). The control group consisted of the remaining 25 infants without sepsis evaluation.

53 eligible VLBW infants were admitted to our NICU during the ten month study period. 50 patients were enrolled in the study after informed consent by the parents. 3 infants were not enrolled because no study personnel was present in the first three days after admission to the NICU. Overall there were 28 male (56.0%) and 22 female (44.0%) infants. 25 patients (50.0%) had at least one episode of confirmed or suspected sepsis. Among these, 7 infants (14.0%) were allocated to the culture positive group, 10 infants (20.0%) to the clinical sepsis group and 8 infants (16.0%) to the indeterminate group. 25 patients (50.0%) comprised the control group. Three of seven infants in the culture positive group suffered from NEC and were allocated according to the positive blood cultures results. The group allocation of the study cohort is depicted in **Fig 1**. Demographic characteristics are summarized in **S1 Table.** Birth weight was significantly higher in the control group compared to the other three groups (p <0.05). Gestational age was significantly lower in the culture positive group compared to the control group (p <0.05) but did not reach statistical significance when compared to the clinical sepsis group and the indeterminate group. There was significant difference in Apgar scores at 5 and 10 minutes and male and female ratio among the groups.

36 patients remained hospitalized in our NICU until postnatal day 28 (72%). In 14 patients study participation was discontinued early because of transferal to hospitals closer to their respective home town (n = 12) or withdrawal of informed consent (n = 2). The mean duration of observation was 20.3 days (median 25; range 1–28 days). The study period (i.e. day 4 until day 28) for any patient consisted of a maximum of 25 days. Consequently, for all 50 patients a maximum of 1,250 days for blood sampling was possible. The sum of all days that the infants remained hospitalized during the study period was 1,046 days (83.7%), taken into account that 14 patients did not complete the entire study period. CD64-index was calculated in 379 blood samples (36.2%) and was available for 41.6%, 49.7%, 43.7%, and 24.7% of possible days during the study period in the culture positive, clinical sepsis, indeterminate, and control group, respectively.

Sepsis evaluation was performed at a median postnatal age of 10 days (range 7–20) in the culture positive, 12 days (range 6–15) in the clinical sepsis and 11.5 days (range 5–18) in the indeterminate group. Blood culture recovered the following organisms: Coagulase-negative staphylococci (four); Enterococcus faecalis (one); and Klebsiella oxytoca (two).

At the time of diagnosis (day 0), three of seven CRP values (43%) and four of seven IL-6 values (57%) were above the respective cut-off in the culture positive group. 6/7 patients (86%) were correctly identified by a combination of either IL-6 or CRP or both above the predetermined cut-off value. In the clinical sepsis group three infants (30.0%) had an increased CRP and one infant (10.0%) an increased IL-6. In the indeterminate group no patient presented with an increased CRP at day 0 and in one patient (12.5%) IL-6 was mildly increased (169 pg/ml).

CD64 indices on day 0 were available for 2 (29%) of the infants in the culture positive group, 3 (30%) in the clinical sepsis group and 6 (75%) in the indeterminate group. In those patients with a CD64 index value on day 0, CD64 index was elevated in 100%, 33.3% and 33.3% in the respective groups.

**Fig 2:**
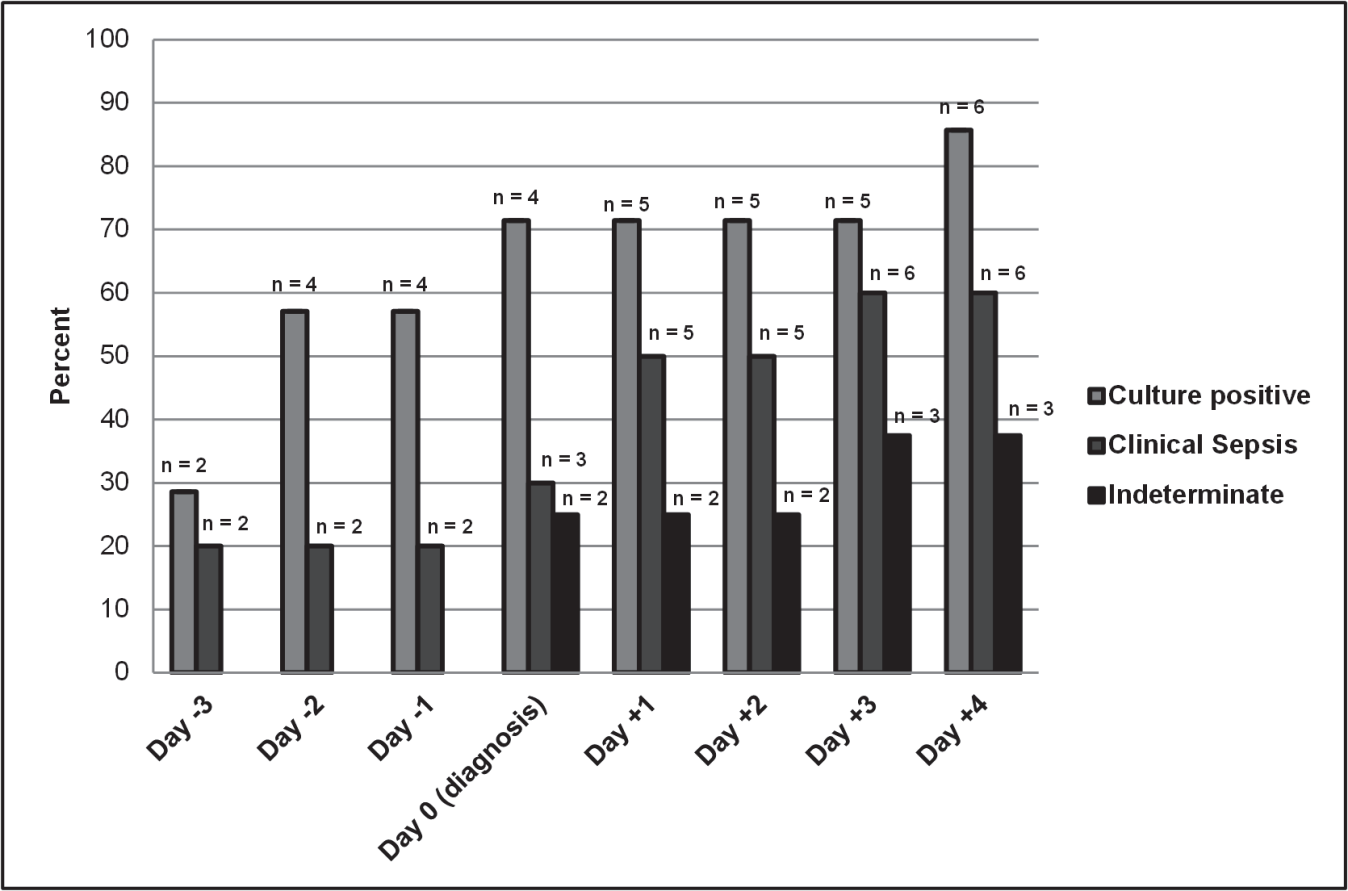
Cumulative incidence of infants with elevated CD64 index values during the observation period. A CD64 index greater than 1.86 was defined as the cut-off value for infection. Day 0 indicates the day of sepsis evaluation (i.e. day of diagnosis). The bars represent the percentage of infants with a positive CD64 index from the beginning of the observation period until the respective day. The differences between groups did not reach statistical significance.

In the culture positive group elevation of CD64 index was noted a median 2 days before diagnosis of sepsis (median at day -2; interquartile range (IQR): day -4 to day 0). This was not observed in the clinical sepsis group (median day 0, IQR day 0) and the indeterminate group (median day 0, IQR day 0). The cumulative proportion of infants with an increased CD64 index value (>1.86) on each day of those in whom CD64 index was determined is shown in **Fig 2**. Overall, there were an increased proportion of infants with elevated CD64 index over the entire observation period (day -4 till day +5). 57% of the patients in the culture positive group had an elevated CD64 index on day -2 and day -1 (n = 4). In contrast, on day -2 and -1 only 20% (n = 2) in the clinical sepsis group and 0% (n = 0) in the indeterminate group had an elevated CD64 index. At least one CD64 index value was available between day 0 and day +5 in five patients in the culture positive group. Two infants had severe neutropenia that resulted in an inappropriate low amount of granulocytes for CD64 index determination between day 0 and day +5.

**Fig 3:**
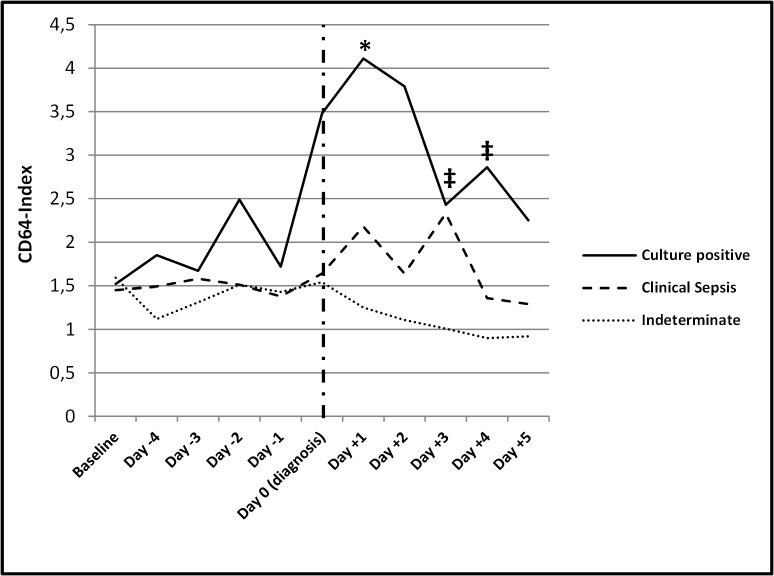
Median CD64 index values during the observation period. Median CD64 index was compared for every day during the observation period (day -4 until day +5) in the respective groups using the Mann-Whitney test. Median CD64 index was significantly higher on day +1 in the culture positive group compared to the clinical sepsis group (p <0.05; *) and significantly higher in the culture positive group compared to the indeterminate group on day +3 and day +4 (p <0.05; ‡). There was no difference in median CD64 index values between the clinical sepsis group and the indeterminate group. The dashed vertical line indicates the day of diagnosis (day 0). The numbers of samples for each data point are reported in Table 3.

Median, minimum and maximum values for CD64 index throughout the observation period are summarized in **S2 Table.** While baseline values were statistically not different (1.52, 1.45 and 1.59 for the culture positive, clinical sepsis and indeterminate group, respectively), the median values throughout the observation period were higher among infants in the culture positive group compared to infants in the clinical sepsis and the indeterminate group **(Fig 3)**. Median CD64 index in the culture positive group was significantly higher compared to the clinical sepsis group on day +1 and compared to the indeterminate group on day +3 and day +4 (p<0.05). Median CD64 index was not significant different between the clinical sepsis and the indeterminate group on each day. The increase from the individual baseline value to the value at any day during the observational period was not significant in each group. However we However, due to the small sample size we did not compare median CD64 index values on the respective days during the observational period to a group specific baseline value.

**Fig 4:**
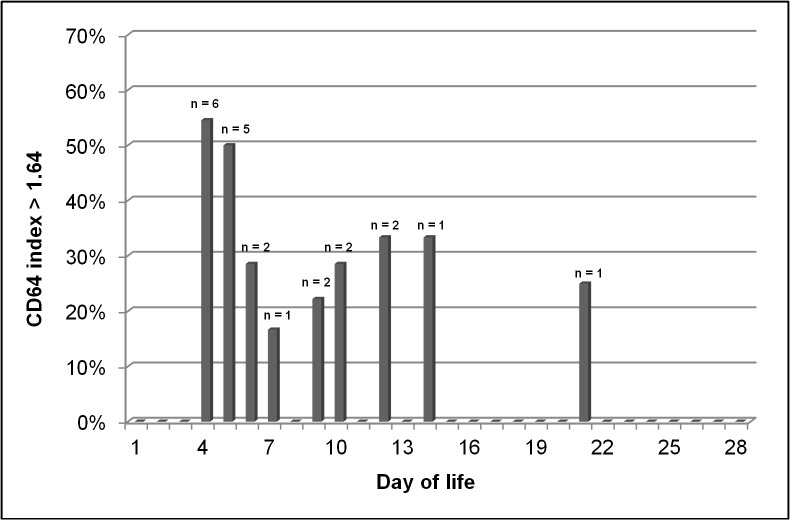
Elevation of CD64 index in asymptomatic infants in the first 28 days of life. Proportion and absolute numbers (n) of infants with elevated CD64 index (>1.86) of all infants in the control group in whom CD64 index had been determined at the respective day between postnatal day 4 and 28 (study period).

A complete blood count was available in the culture positive group, the clinical sepsis group and the indeterminate group on 8, 11, and 6 days, respectively. Using a cut-off value of 150,000 platelets/mm^3^, the cumulative incidence of thrombocytopenia on day -1 was 30% (3/10 infants) in the clinical sepsis group and 0% in the culture positive group and the indeterminate group. Neutropenia (absolute neutrophil count < 7,500/mm^3^) was present on day -1 in 20% (2/10) of infants in the clinical sepsis group, in 14.3% (1/7) of infants in the culture positive group and 12.5% (1/8) of infants in the indeterminate group. Among the 25 infants in the control group, the median CD64 index value was 1.1 (range 0.62–5.3). Median CD64 index in the control group was elevated on day 4 (1.92) and 5 (1.92) after birth but decreased to an average median CD64 index of 1.08 (range 0.68–1.46). **Fig 4**demonstrates the amount of patients in the control group with a CD64 index above the cut-off value of 1.86 on each day during the study period. 12 episodes of CD64 activation were identified in 10 patients (40%) in the control group. The median duration of all inflammation episodes was one day (range 1–10 days). 67% (8/12) of episodes began on postnatal day 4 or 5 (median 4.5; range 4–21). **S3 Table** summarizes details of all 12 episodes. An association between maternal inflammation and an elevated CD64 index was present for 4 of the 8 early episodes (i.e. beginning on postnatal day 4 or 5). For three episodes (triplets) no information on maternal white blood count or CRP was available but the reason for delivery was preterm contractions. One episode was associated with a large grade 3 intraventricular hemorrhage (IVH). In three of four late episodes no association was possible. One episode was strongly associated with initiation of mechanical ventilation. 20 of the 25 infants in the control group did not have increased CD64 index values between postnatal day 6 and 28. In the remaining five patients CD64 index decreased during the study period below the cut-off value.

## Discussion

Early diagnosis of sepsis and infection in VLBW infants is paramount, because this significantly impacts mortality and morbidity [1]. Many studies investigated the potential role of different cytokines or biomarkers as a tool for early diagnosing neonates with sepsis [26]. However, the vast majority of studies focused on infants already presenting with signs of infection and sepsis. Identifying patients with bacteremia before they actually show clinical signs of infection could lead to earlier treatment and might potentially reduce mortality [5]. In the current study we used CD64 index as a screening parameter in 50 VLBW infants during the first 28 days of life. For this purpose an extra blood sample for CD64 analysis was collected whenever blood sampling for other reasons was necessary (e.g. blood gas analysis, routine blood chemistry). Our data demonstrates higher values for CD64 index on the day of diagnosis in patients with culture proven sepsis, compared to patients with clinical sepsis and with suspicion of sepsis (day 0: median of 3.48, 1.64 and 1.54 respectively). Furthermore, CD64 index was above a predefined cut-off value (CD64 index >1.86) in 4 of 7 patients (57.1%) with culture proven sepsis at least two days before the initiation of antibiotic treatment. In contrast to the findings in the culture positive group, none of the infants in the indeterminate group had an elevated CD64 index before initiation of antibiotic therapy.

CD64 is continuously expressed at a very low level on the surface of neutrophil leukocytes in absence of bacterial stimulation. During bacterial sepsis the expression of CD64 is significantly increased [19,27]. CD64 activation is different from the LPS-induced pro-inflammatory cytokine pathway. CD64 expression on granulocytes is induced by IgG, interferon gamma or granulocyte colony-stimulating factor [11]. Following contact with an antigen the antigen-IgG complex binds to CD64 and cross-link to each other. This process further increases CD64 expression on neutrophils. CD64 activation promotes phagocytosis and intracellular killing. Low concentrations of IgG in the serum of extremely low gestational age neonates, when transplacental IgG transfer was not sufficient, was considered as a possible problem of the utility of CD64 during sepsis in these patients. However, Several studies have investigated the diagnostic power of absolute CD64 count and CD64 index as successful biomarkers for term and preterm infants with EOS and LOS [3,13,14,15,16,17,18,19,22,25,28,29]. Recently, one study was published using CD64 count as a surveillance marker for LOS/NEC in VLBW infants [6]. Routine measurement of absolute CD64 expression detected infection a mean of 1.5 days before clinical presentation.

The measurement of neutrophil CD64 by flow cytometry is a rapid assay providing the clinician with prompt results. After the blood sample is transferred to the laboratory the entire processing time to calculate CD64 index takes approximately two to three hours. Processing time and round-the-clock availability are important requirements for the clinical practicability of a parameter, but we don’t see these drawbacks for CD64 index. It has also been demonstrated that routine hematology analyzers can perform CD64 determination with reliable results [30]. In the present study, to avoid cell apoptosis flow cytometry was performed within 24 hours. There has been some concern about the comparability of the results of existing studies because CD64 measurement has been performed on different flow cytometers [30]. The use of the QuantiCalc software for an automated analysis of the CD64 index has been postulated to be superior to the absolute neutrophil CD64 count [13,25,30].

In a previous study, Ng et al. reported a higher sensitivity and negative predictive value of IL-6 compared to CRP for diagnosing sepsis in VLBW infants [31]. They found CRP to be of lower sensitivity for diagnosis of infection, but of increased sensitivity and specificity when measured 24 and 48 hours after diagnosis [31]. CRP was helpful for the evaluation of the course of an infection and for monitoring the efficacy of antibiotic therapy [32,33]. For diagnosis of bacterial infection among preterm infants, the combination of IL-6 and CRP had a high sensitivity and specificity [31]. However, it is important to remember that IL6 has a short half-life, and that the combination of IL6 and CRP may lead to a diagnostic gap when IL6 already decreased to normal and CRP is not yet increased. CD64 is increased early in the course of infection and remains stable for several days [28]. In our study, CRP and IL-6 were increased at day 0 in the culture positive group in 42.9% and 57.1%, respectively. In the clinical sepsis group only 30% and 10% of patients had elevated CRP and IL-6, respectively. In the indeterminate group, no patient had an increased CRP and only one patient (12.5%) had a mildly increased IL-6 at day 0.

Thrombocytopenia and neutropenia occur frequently during sepsis in preterm infants, but individually they lack sensitivity and specificity [34]. Considered together with other parameters, an abnormal platelet and neutrophil count can increase the diagnostic performance [35]. In a study investigating the role of neutrophil CD64 in combination with hematologic criteria in neonatal sepsis, Streimish et al. demonstrated a sensitivity, specificity, positive predictive value and negative predictive value for thrombocytopenia and neutropenia to diagnose late-onset sepsis of 56%, 73%, 21%, and 93%, and 59%, 22%, 9%, and 81%, respectively [18]. Neutrophil CD64, in combination with the absolute neutrophil count, had the highest sensitivity (91%) to diagnose culture proven sepsis [18]. CD64 in combination with the absolute band count had the highest specificity (93%) [18]. Due to the small number of patients and values in the respective groups in our study, we did not combine CD64 index values with hematologic criteria. Our study protocol required a daily surveillance of clinical signs of infection or sepsis. Therefore, we can emphasize that patients in the respective groups were asymptomatic during the days preceding the day of diagnosis and that treatment was not started with delay. Using 80 bpm instead of 100 bpm as definition for bradycardia may possibly result in a later diagnosis of sepsis. For two reasons we do not believe that this approach may have excluded patients. First, at least two clinical signs suspicious for infection had to be present and bradycardia rarely occurs as a sole clinical sign during the course of infection. Second, infants admitted to our NICU were closely monitored for signs of infection as part of our routine clinical care and misdiagnosing an infection in patients only because of the threshold for bradycardia seems unlikely. However, to our knowledge no study has been published comparing different thresholds for bradycardia in preterm infants with sepsis or infection. Heart rate abnormalities (i.e. bradycardia and tachycardia) are unspecific for infection in VLBW infants. In a recent study by Bekhof et al, the most important clinical signs suggestive of late onset sepsis in preterms were increased respiratory support, capillary refill, grey skin and a central venous catheter while other clinical signs were rather unspecific [36].

40% of infants (n = 10) in our control group, consisting of patients without evaluation for suspected sepsis during the first 28 days of life, had at least one episode of inflammation (i.e. at least one elevated CD64 index) during the entire study period. The majority of these values (63.6%) occurred between day 4 and 7. Maternal inflammation or infection, defined as clinical signs of infection and increased CRP or leukocytosis, was likely present in the majority of early occurring episodes. In the current study, maternal betamethasone and maternal antibiotics were administered to mothers of 38 (76%) and 41 (82%) infants, respectively. Previous studies demonstrated that both could possibly alter maternal and neonatal inflammatory response [37,38,39,40,41]. Considering this, our definition of maternal inflammation should be used cautiously. Although maternal steroids and antibiotics could be relevant for the current study, this issue has not been investigated for CD64 expression in premature infants.

Laboratory values were missing for a mother of triplets, but the indication for caesarean section was preterm contractions and tachycardia of one infant. All infants were treated with antibiotics for three to five days and the highest CRP value among these triplets was 6.2 mg/l. Another child developed prenatally a large intraventricular hemorrhage (grade III), that was associated with maternal anticoagulation for homozygote factor V Leiden thrombophilia.

In summary, the cut-off of 1.86 does not seem to be appropriate in the first week of life, considering that more than 50% of infants in the control group had values >1.86 until day 7.

We were not able to provide helpful information about late occurring episodes of inflammation. One episode was associated with intubation and mechanical ventilation in an infant with respiratory failure. For the remaining three late episodes chart review did not reveal further information. Only 9.9% of the CD64 index values between day 8 and 28 in the control group were above the cut-off value of 1.86. Lam et al. described similar findings in their study among 146 preterm infants and concluded that asymptomatic CD64 activation would result in an 41% increase of sepsis evaluations [6].

Systemic inflammation is an important contributor to neonatal morbidity [42]. The potential neurotoxic effect of inflammation and the role in the etiology of white matter disease in preterm infants has been described in detail [43,44]. Non-infectious inflammation in preterm infants has been associated with several invasive therapies such as mechanical ventilation [45], parenteral nutrition [46,47] and oxygen supplementation [46,48]. Cai et al demonstrated in an animal model of neonatal brain injury the detrimental effect of intracerebral injection of the pro-inflammatory cytokine Interleukin-1b in the absence of infection [49]. Although these studies shed some light on the role of non-infectious inflammation in preterm infants, more studies are needed for a broader understanding.

Due to the relatively low specificity, CD64 is probably not the most desirable biomarker for screening infants at risk for LOS. However, using multiplex immunoassays a broad variety of inflammatory markers can be analyzed with small amounts of blood [50]. This could reveal insights into inflammatory processes in septic VLBW infants in the time before they demonstrate with symptoms of infection.

### Limitations

The relatively small sample size is certainly a limitation of our study, especially because infants were allocated to 4 different study groups. However, this approach was used to reflect the clinical situation in a NICU setting, where patients either have culture positive sepsis, clinical sepsis with strong biochemical and clinical evidence for bacterial infection, or present with signs and symptoms of sepsis but are treated with antibiotics only for a few days. Because of the small number of patients and large confidence intervals in every group we did not report cut-off values, sensitivity or specificity. The cut-off values for CD64 index vary between studies and are also influenced by prematurity [17,18,19,22,25,51]. We used a cut-off value of 1.86 because this has been described previously with the highest sensitivity and specificity [25]. There are no cut-off values for CD64 index for the days before clinical manifestation of sepsis, because no study investigated its role as screening marker for neonatal sepsis. Therefore, there might be a better cut-off value for CD64 index in the presence of infection than 1.86. This might especially be true during the first week of life when there is still an impact of maternal infection and an increased need for invasive therapies. The study protocol allowed collection of blood for CD64 determination only during routine blood sampling and therefore the exact daily kinetics of CD64 remains uncertain. Additionally, our NICU policy to perform blood gas analysis in VLBW infants was restrictive and in stable patients this was done once or twice a week resulting in a relatively low amount of samples during the study period. Another limitation concerns neutropenia. Two infants in the culture positive group had severe neutropenia during the episode of sepsis, resulting in an inappropriate low amount of granulocytes for CD64 index determination. In our study, the collected 50 μl of whole blood might be a critical amount for infants with a low neutrophil count. The Leuko64 kit requires 50,000 neutrophils for a valid CD64 count. This limitation has been described by other study groups and should be taken in account when using CD64 as a biomarker for sepsis [13,52].

## Conclusion

In the current study we were able to demonstrate that a substantial amount of patients with culture proven sepsis had increased CD64 index values during the days before clinical signs of sepsis were recognized. This finding is similar to other studies with a comparable design [5,6].

Although the approach of routine measurements of biomarkers for infection seems promising, the optimal biomarker for sepsis and infection remains unknown. Whether daily screening for infection could decrease the burden of sepsis complications or the duration of antibiotic treatment in VLBW infants should be investigated in a large cohort.

Based on our results we see potential for further research on two topics. First, a large prospective trial is needed to investigate the optimal biomarker for early identification of LOS in the preterm population using multiple biomarkers. Second, more research effort should be focused on the role of non-infectious inflammation on neonatal morbidity (e.g. neurocognitive outcome, bronchopulmonary dysplasia).

## Supporting Information

S1 Table Patient characteristics of the study groups. Gestational age and birth weight are described as mean ± standard error of the mean. Apgar-Score are reported as median with range. The p-value describes statistical significance between the culture positive and the control group.
(XLSX)

S2 Table Median, maximum and minimum values for CD64-index during the observation period. (XLSX)

S3 Table Characteristics of episodes of inflammation in the control group. * Maternal inflammation was defined as an elevated white blood count and/or an elevated CRP in maternal blood and clinical signs of infection within 3 days prior or after delivery. Abbr.: N/A = Information not available; IUGR = intrauterine growth retardation; pPROM = preterm premature rupture of membranes.
(XLSX)

S4 Table CD64 index observation period. This file demonstrates single values for CD64 index during the observation period in the culture positive, clinical sepsis, and indeterminate group.
(XLSX)
